# Cytosolic BNIP3 Dimer Interacts with Mitochondrial BAX Forming Heterodimers in the Mitochondrial Outer Membrane under Basal Conditions

**DOI:** 10.3390/ijms18040687

**Published:** 2017-03-23

**Authors:** Ulrike B. Hendgen-Cotta, Sonja Esfeld, Katharina Rudi, Ilkka Miinalainen, Johann P. Klare, Tienush Rassaf

**Affiliations:** 1 Department of Cardiology and Vascular Medicine, West German Heart and Vascular Center, Medical Faculty, University Hospital Essen, Hufelandstr. 55, 45147 Essen, Germany; sonja.esfeld@uk-essen.de (S.E.); tienush.rassaf@uk-essen.de (T.R.); 2 Department of Physics, University of Osnabrück, Barbarastr. 7, 49069 Osnabrück, Germany; katharina.rudi@uni-osnabrueck.de (K.R.); jklare@uni-osnabrueck.de (J.P.K.); 3 Biocenter Oulu, University of Oulu, Aapistie 5A, 90220 Oulu, Finland; ilkka.miinalainen@oulu.fi

**Keywords:** BNIP3, BAX, cell death, heterodimerization, homodimerization, mitochondria

## Abstract

The primary function of mitochondria is energy production, a task of particular importance especially for cells with a high energy demand like cardiomyocytes. The B-cell lymphoma (BCL-2) family member BCL-2 adenovirus E1B 19 kDa-interacting protein 3 (BNIP3) is linked to mitochondrial targeting after homodimerization, where it functions in inner membrane depolarization and permeabilization of the mitochondrial outer membrane (MOM) mediating cell death. We investigated the basal distribution of cardiac BNIP3 in vivo and its physical interaction with the pro-death protein BCL2 associated X, apoptosis regulator (BAX) and with mitochondria using immunoblot analysis, co-immunoprecipitation, and continuous wave and pulsed electron paramagnetic resonance spectroscopy techniques. We found that BNIP3 is present as a dimer in the cytosol and in the outer membrane of cardiac mitochondria under basal conditions. It forms disulfide-bridged, but mainly non-covalent dimers in the cytosol. Heterodimers with BAX are formed exclusively in the MOM. Furthermore, our results suggest that BNIP3 interacts with the MOM directly via mitochondrial BAX. However, the physical interactions with BAX and the MOM did not affect the membrane potential and cell viability. These findings suggest that another stimulus other than the mere existence of the BNIP3/BAX dimer in the MOM is required to promote BNIP3 cell-death activity; this could be a potential disturbance of the BNIP3 distribution homeostasis, namely in the direction of the mitochondria.

## 1. Introduction

BCL-2 adenovirus E1B 19 kDa-interacting protein 3 (BNIP3) is a member of the B-cell lymphoma (BCL-2) subgroup of BCL-2 homology 3 (BH3)-only proteins and mediates cell death resembling the characteristics described for apoptosis and necrosis. BNIP3 is involved in disturbing mitochondrial function by perturbation of the electrochemical gradient across the mitochondrial inner membrane (MIM) as shown in heart like (HL-1) cells in the scope of hypoxia [1,2] and in Huntington disease [3]. Dimerization and localization of BNIP3 to the mitochondrial outer membrane (MOM) after cellular stress is also considered to cause mitochondria dysfunction and, subsequently, cause non-apoptotic and apoptotic cell death [1,4,5]. This feature is suggested to play a wide role in the pathogenesis of numerous diseases including heart failure [6,7], stroke [8], and cancer [9,10,11]. Thereby, death signals seem to converge on BCL2 associated X, apoptosis regulator (BAX) a BCL-2 protein or voltage-dependent anion channel (VDAC), which is triggered by BNIP3 and precipitates membrane permeabilization of the MOM, sometimes leading to mitochondrial swelling [12,13,14]. This allows release of apoptogenic factors localized within the mitochondrial intermembrane space like cytochrome *c* and endonuclease G [12,13,14]. BNIP3 contains a transmembrane domain at its C-terminus that should be responsible for dimerization, MOM targeting, and pro-death activity [12,15,16,17]. For the latter property, a conserved cysteine residue near the N-terminus facing the cytosol also seems to be necessary for stabilization of the homodimer [12,17]. Numerous putative binding partners are published for BNIP3-forming heterodimers, namely VDAC, OPA1, and the anti-apoptotic family members BCL-2 and BCL-X_L_ [17,18,19], but a binding to BAX has not been described until now. Intriguingly, the common feature of all aforementioned signaling pathways is targeting of the mitochondrion by BNIP3. How BNIP3 interacts with mitochondria under basal conditions still awaits clarification. Gaining insight into the distribution and physical interactions of BNIP3 and its impact on MIM potential and cell death in physiology will be of great value in understanding the molecular mechanisms that drive BNIP3-mediated cell death in pathology. To deconvolve the binding of cardiac BNIP3 to mitochondria, we first focused on the in vivo localization and the oligomeric status of BNIP3 using cell fractionation, electron microscopy (EM), immunoblotting, and flow cytometry analysis. Furthermore, we analyzed BAX as a potential binding partner via immunoprecipitation. We applied spin labeling and electron paramagnetic resonance (EPR) spectroscopy [20] with recombinant murine rBNIP3 and rBAX and isolated mitochondria from mouse hearts to investigate the underlying mechanism.

## 2. Results and Discussion

### 2.1. BNIP3 Forms Different Dimers in the Cytosol and in the MOM in Mouse Heart Cells

**Figure 1:**
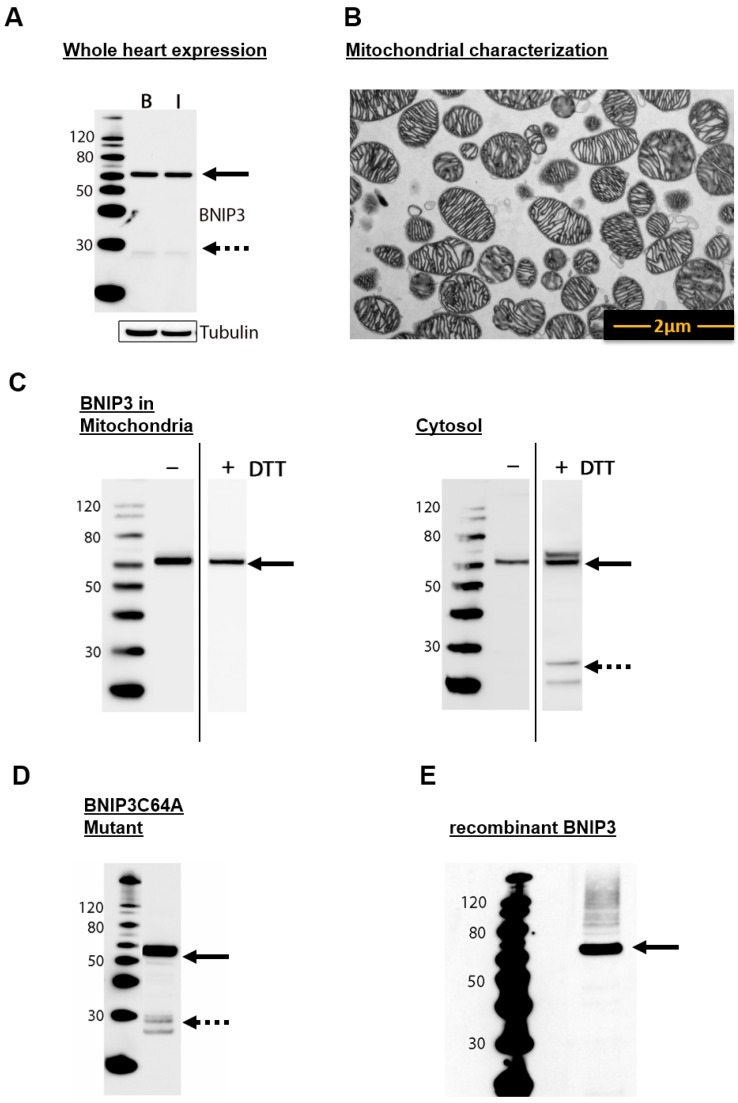
Localization of BCL-2 adenovirus E1B 19 kDa-interacting protein 3 (BNIP3). (**A**) Immunoblot analysis of BNIP3 under reducing conditions in whole mouse heart under basal conditions (B) and after 30 min of myocardial ischemia (I) (*n* = 9). BNIP3 could be detected in a 60 kDa dimeric form and a 30 kDa monomeric form; (**B**) Examination of isolated mitochondria from whole mouse hearts using electron microscopy (magnification 4800×) (*n* = 3). Pure mitochondria could be isolated in high concentrations; (**C**) Immunoblot analysis of BNIP3 in separated mitochondrial and cytoplasmic fractions from whole mouse hearts under non-reducing and reducing (50 mM dithiothreitol) conditions (*n* = 3); (**D**) Immunoblot analysis of BNIP3 mutant C64A under reduced conditions (*n* = 3); (**E**) Immunoblot analysis of recombinant murine BNIP3 under reduced conditions (*n* = 3). Black arrows mark the dimeric and dashed arrows the monomeric protein form.

In accordance with previous observations in rat heart homogenates [12] and non-cardiac cell cultures [17,21], on sodium dodecyl sulfate polyacrylamide gel electrophoresis (SDS-PAGE), BNIP3 in mouse heart homogenates migrates as a ~30 kDa monomer and a SDS- and dithiothreitol (DTT)-resistant ~60 kDa dimer species (Figure 1A). Different cell types exhibit diverging concentrations of BNIP3 under basal conditions [1,5,21,22]. Whereas cultured neonatal cardiomyocytes lack detectable BNIP3 expression [5,21], which is up-regulated by exposing the cells to hypoxia, high-level expression of BNIP3 protein is observed in whole adult mouse heart homogenates under physiological conditions (Figure 1A). Furthermore, the expression level and the formation of non-covalent dimer species remain unchanged in mice subjected to 30 min of ischemia in vivo (Figure 1A). To validate the abundance and spatial distribution of BNIP3 we separated cytosol and mitochondria from homogenized mouse heart cells. The mitochondrial fraction was examined by electron microscopy (Figure 1B). Contrary to previous observations that BNIP3 is found solely associated with the mitochondria-enriched heavy membrane fraction of non-perfused rat heart lysates [5], immunoblot analysis of the cell fractions reveals that BNIP3 is present in both the mitochondrial and cytosolic fractions of adult mouse heart homogenates (Figure 1C). Interestingly, BNIP3 appears to occur solely in the form of dimers (60 kDa) in mitochondria and in the cytosol (Figure 1C) under normal (non-reducing) conditions. Treatment with DTT and heat (50 mM DTT, 5 min at 95 °C, reducing conditions) resulting in cleavage of possible disulfide bridges involving the conserved cysteine residue at position 58 (Cys64 in human BNIP3) shows no influence on the oligomeric state of mitochondrial BNIP3, suggesting that the dimer is tightly held together by non-covalent interactions that render the complex SDS-resistant. In vitro experiments with BNIP3 mutants with the conserved cysteine residue mutated to alanine confirmed that formation of BNIP3 heterodimers occurs independently of disulfide bonds (Figure 1D). In the cytosol, BNIP3 also exists as SDS-resistant non-covalent dimers and appears to form a few covalent (disulfide-bridged) dimers (cf. Figure 1C). The partial DTT-sensitivity of cytosolic BNIP3 is reflected in the weak appearance of a monomeric form after reduction (Figure 1C, left panel). Two possible explanations for the observed DTT-resistant (non-covalent) and DTT-sensitive (covalent) BNIP3 dimers are: (i) the two forms represent reduced and less oxidized BNIP3 homodimers, owing to less but existent levels of reactive oxygen species under physiological conditions. In vitro experiments with rBNIP3 (Figure 1E) clearly show that BNIP3 forms a 60 kDa homodimer in solution under reducing conditions, strongly suggesting that the non-covalent DTT-resistant dimers found in the cytosol include a BNIP3 homodimer [12]; additionally, (ii) the DTT-resistant dimer could be a heterodimer.

**Figure 2:**
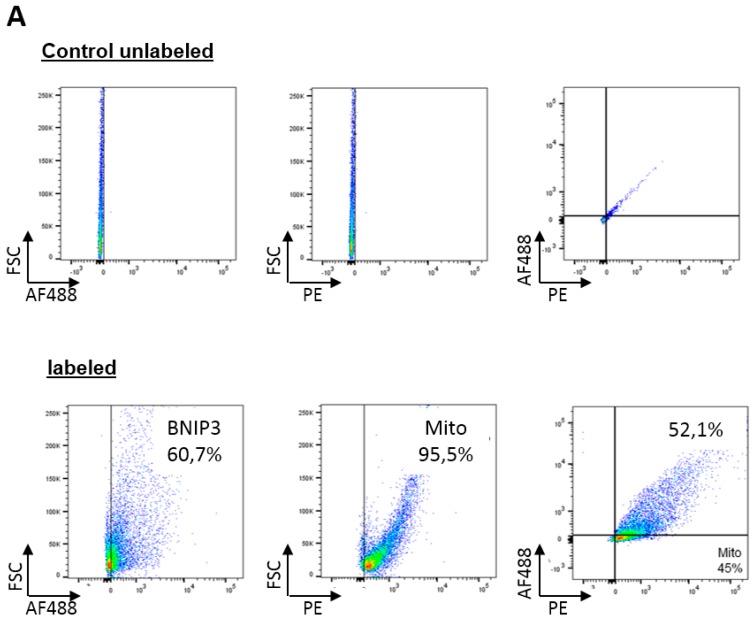

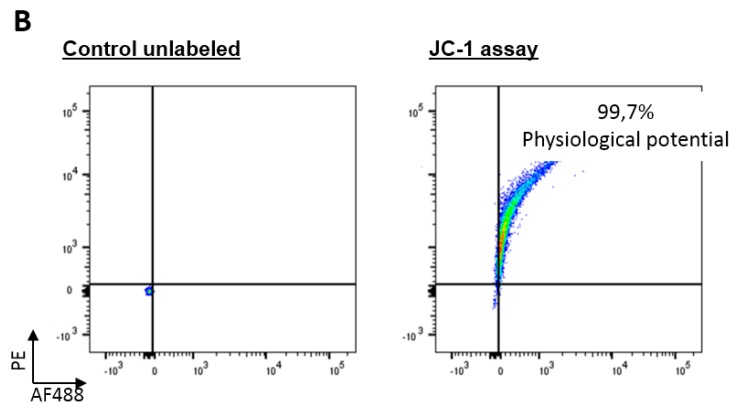
Impact of BNIP3 on mitochondria and cell integrity. (**A**) Flow cytometry analyses of isolated mouse heart mitochondria. The upper dot plots show the particular unlabeled controls. BNIP3 were labeled using AF488 and mitochondria using MitoTracker (Phycoerythrin (PE)-labeled) (*n* = 3). A total of 52.1% of mitochondria showed double positive; (**B**) Measurement of the mitochondrial inner membrane potential from isolated mouse heart mitochondria under basal conditions using 5,5′,6,6′-tetrachloro-1,1′,3,3′-tetraethylenbenzimidazolylcarbocyanin iodid (JC-1) fluorescence-activated cell sorting (FACS) assay. The left dot plots show the unlabeled controls and the right dot plots the labeled mitochondria (*n* = 5).

To evaluate the exact localization of BNIP3 in mitochondria, we performed surface marker flow cytometry analysis with isolated mitochondria (Figure 2A). Binding of the membrane-potential-dependent MitoTracker probe (Figure 2A, left panel) confirmed the intactness of at least ~95.5% of the mitochondria. The determination of BNIP3 (Figure 2A, center and right panel) revealed that at least half of the mitochondria is BNIP3 positive. BNIP3 is localized on the surface of mitochondria and integrated into the MOM, as it is accessible for immunoblotting with intact mitochondria (Figure 2A, center and right panel). To gain insight into whether the localization of BNIP3 in mitochondria affects the MIM potential, we used the 5,5′,6,6′-tetrachloro-1,1′,3,3′-tetraethylbenzimidazolcarbocyanine iodide (JC)-1 assay. Fluorescence-activated cell sorting (FACS) analyses revealed that 99.7% of mitochondria exhibit a physiological MIM potential under basal conditions (Figure 2B). Considering that a minimum of about half of the mitochondria are BNIP3 positive, this indicates that neither the presence nor the absence of BNIP3 has an effect on the MIM potential under basal conditions. Furthermore, staining of whole heart slices with 2,3,5-triphenyltetrazoliumchlorid (TTC) for demarcation of viable and non-viable myocardium exhibits no cardiac injury (data not shown).

### 2.2. BNIP3 Interacts with Mitochondrial BAX in the MOM In Vivo under Basal Conditions

**Figure 3:**
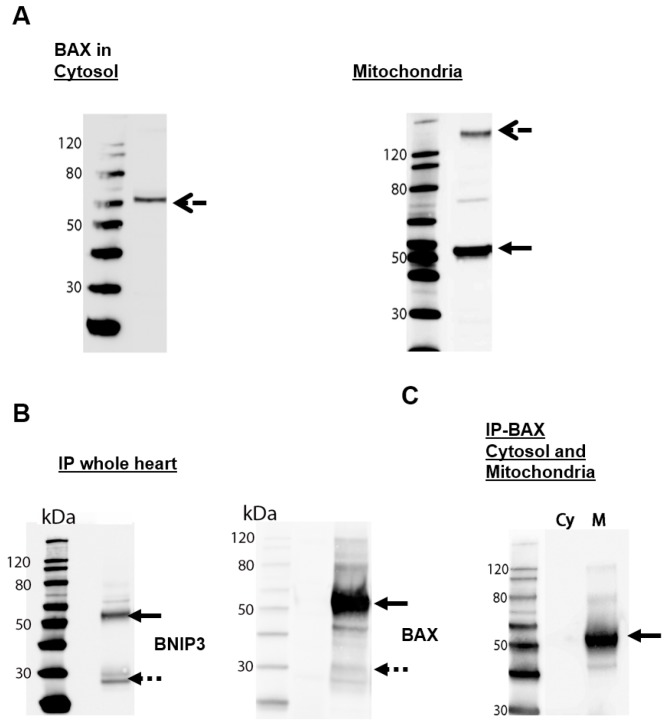
BNIP3 interacts with BAX in murine heart cells. (**A**) Immunoblot analysis of BAX in separated cytosolic and mitochondrial fractions from whole murine hearts under reduced conditions (*n* = 3); (**B**) Co-immunoprecipitation reveals an interaction of BNIP3 and BAX. Protein lysates from whole mouse hearts were precipitated with BAX (**left** panel) and BNIP3 (**right** panel) labeled dynabeads and analyzed using an immunoblot technique to detect the binding partner (*n* = 5); (**C**) Investigation of BNIP3 and BAX interactions in separated cytosolic (Cy) and mitochondrial (M) fractions using co-immunoprecipitation with BAX antibody (*n* = 3). Rude dashed arrows mark the oligomeric, black arrows the dimeric and dashed arrows the monomeric protein form.

To evaluate the possibility that some of the observed BNIP3 dimers are heterodimers formed with BAX, we first determined the presence of BAX in cytosolic and mitochondrial fractions. In vivo, BAX is present in the cytosol and in mitochondria isolated and separated from mouse hearts (Figure 3A). Immunoblotting analysis revealed that cytosolic BAX is present as a 75 kDa oligomer, whereas in the mitochondrial fraction, BAX predominantly forms 60 kDa dimers together with small amounts of ~170 kDa oligomers (Figure 3A). We then performed co-immunoprecipitation analysis to discover interactions between BNIP3 and BAX in vivo under basal conditions using whole mouse hearts as well as separated cytosol and mitochondria. We isolated the proteins from heart homogenates, precipitated BAX and the interaction partners via dynabeads, and determined the presence of BNIP3 by immunoblotting analysis (Figure 3B, left panel). To confirm the results, we also performed the experiment in the opposite order, precipitating BNIP3 and staining against BAX (Figure 3B, right panel). Both experiments clearly reveal BAX and BNIP3 to be binding partners. In both cases, the major species observed on the blots have a molecular weight of ~50–60 kDa, confirming that reduction-resistant (reductive conditions, see Materials and Methods) BNIP3/BAX heterodimers are formed in vivo. The detection of monomeric species (~25 kDa) may indicate a limited stability of the BNIP3/BAX dimer under the experimental conditions (SDS). Using separated cytosolic and mitochondrial fractions for immunoprecipitation of BAX and BNIP3 (Figure 3C) it becomes apparent that BNIP3/BAX heterodimers are exclusively formed in mitochondria. Consequently, the absence of BNIP3/BAX dimers in the cytosol (Figure 2C) indicates that cytosolic BAX do not interact with cytosolic BNIP3. The latter species most likely represent a mitochondria-bound fraction of the DTT-sensitive oligomer found in the cytosol. Taken together, our results indicate that at least a fraction of the reduction-resistant dimers observed in the MOM are BNIP3/BAX heterodimers. Based on the experimental results described above, we cannot exclude the additional presence of BNIP3 homodimers in the MOM.

### 2.3. BNIP3 Homodimers Dissociate upon Interaction with BAX and Mitochondria

**Figure 4:**
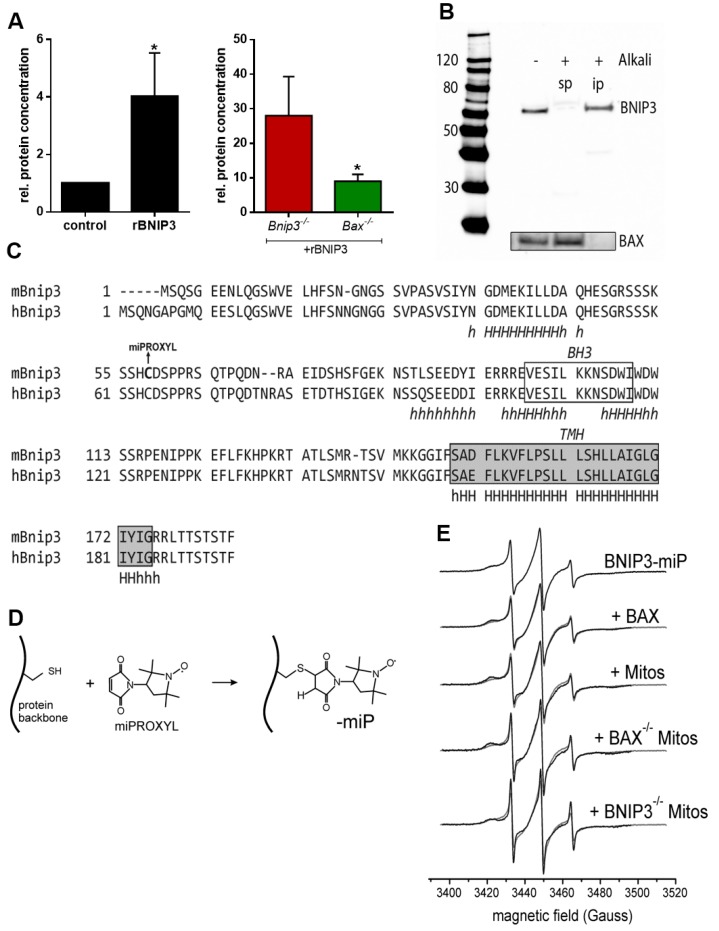
BNIP3 interacts with mitochondria via BAX. (**A**) Incubation of mitochondria with recombinant BNIP3 (rBNIP3) leads to a significant increase of the BNIP3 protein concentration in mitochondria (**left** panel; one-tailed ANOVA; *n* = 3; * *p* = 0.0124). Incubation of *Bnip3^−/−^* mitochondria with rBNIP3 also leads to a strong increase of the BNIP3 protein content in the organelles, whereas rBNIP3 incorporates to a significantly lower extent into *Bax^−/−^* mitochondria (**right** panel). One tailed ANOVA (*n* = 2/3; *p* = 0.0268); (**B**) Immunoblot analyses after alkali extraction of mitochondria show that BNIP3 is an integral membrane protein. Contrarily, BAX is found in the soluble protein fraction after alkali treatment (sp = soluble proteins; ip = integral proteins); (**C**) Primary sequences of murine (m)BNIP3 and human (h)BNIP3 (aligned with ClustalW 1.83) with secondary structure predictions (HHpred). The locations of the Bcl-2 homology 3(BH3) domain and the transmembrane helix (TMH) are indicated. Spin labeling of mBNIP3 at the native Cys58 has been carried out with 3-Maleimido-PROXYL (miP); (**D**) Reaction of the maleimide spin label with a cysteine side chain; (**E**) X band continuous wave electron paramagnetic resonance spectra for BNIP3-wild-type-miP (from **top** to **bottom**) alone, in the presence of BAX and incubated with isolated mouse heart mitochondria from wild-type (wt), *Bax*^−/−^ and *Bnip3^−^*^/−^ mice. All spectra have been recorded at room temperature (298 K) (*n* = 2).

To investigate the interaction of BNIP3 with mitochondria, we incubated recombinant BNIP3 (rBNIP3) with mitochondria from wild-type (wt), *Bnip3^−/−^* and *Bax^−/−^* mice and performed quantitative analysis of the mitochondrial BNIP3 content by immunoblotting (Figure 4). rBNIP3 incorporates into wt mitochondria, increasing the overall content ~4-fold. As expected, rBNIP3 incorporated strongly into *Bnip3^−/−^* mitochondria (Figure 4A). *Bax^−/−^* mitochondria showed a significantly lower incorporation of rBNIP3 (Figure 4A). Nevertheless, incorporation of rBNIP3 still occurs, presumably via interaction with other MOM constituents, e.g., VDAC and/or in the form of homodimers.

We further tested whether BNIP3 really integrates into the mitochondrial membrane, as suggested by the presence of a putative transmembrane domain at its C-terminus [15,17]. After alkali extraction of proteins from mitochondria, BNIP3 remained tightly associated with the mitochondrial membrane (Figure 4B), indicating membrane-insertion of the protein. Remarkably, as reported before [5], BAX detaches from the membrane and is found in the soluble fraction after alkali treatment, indicating that it is only loosely attached to the MOM (Figure 4B). This strongly suggests that the BNIP3/BAX dimer inserts into the MOM only via BNIP3, with BAX non-covalently attached to it. 

To gain insights into the structural properties of the BNIP3 interactions, we utilized the native cysteine residue in wild-type (wt) BNIP3 (C58, Figure 4C) for covalent labeling with a nitroxide spin label (3-maleimido-PROXYL, miPROXYL; Figure 4D), and conducted continuous wave (cw) electron paramagnetic resonance (EPR) and double-electron-electron resonance (DEER) inter spin distance measurements with spin labeled rBNIP3 (rBNIP3-miP). We decided to use a maleimido functionalized nitroxide spin label compound, as the resulting covalent C-S bond between the label side chain and the protein (Figure 4D) is more resistant to potential reduction and cleavage under certain experimental conditions (e.g., in the experiments with isolated mitochondria, see below) compared to the most commonly used methanethiosulfonate spin labels that are coupled to the protein via a disulfide bridge. We obtained labeling efficiencies for rBNIP3 of ~70%. Noteworthy, using the conserved cysteine residue in BNIP3 for labeling precludes the putative oxidized (disulfide bridged) form of the homodimer from being studied. Figure 4E (top panel) shows the X band cw EPR spectrum obtained for rBNIP3-miP (3-Maleimido-PROXYL) in solution. The EPR spectral shape at room temperature reports about the reorientational freedom of the spin label side chain. Narrow EPR lines and a small apparent hyperfine splitting (the distance between the EPR lines on the magnetic field axis) indicate a fast reorientational motion of the label side chain. With increasing immobilization of the spin label (for example, caused by contacts to neighboring protein side chains), increasing EPR line widths and hyperfine splittings are observed. The spectral shape observed for rBNIP3-miP is characteristic for a partially immobilized spin label side chain covalently bound to a protein. Incubation of rBNIP3-miP (15 µM) with rBAX (100 µM) in a phosphate-buffered saline (PBS) buffer (Figure 4E, 2nd panel) leads to small but reproducible spectral changes, indicating an altered spin label mobility caused by structural rearrangements in the vicinity of the label side chain upon interaction with BAX. Therefore, the EPR spectrum for BNIP3-miP + BAX is expected to exhibit contributions from all those species, although we used ~7-fold excess of rBAX over rBNIP3-miP to drive the equilibrium toward the BNIP3/BAX heterooligomers. Consequently, the EPR spectrum for a “pure” BNIP3/BAX solution dimer might differ more strongly from that of the BNIP3-miP homodimer than suggested by the minor spectral changes observed.

Strikingly, incubation of rBNIP3-miP (15 µM) with isolated mouse heart mitochondria (60 µg/µL) (Figure 4E, 3rd panel) leads to very similar and slightly more expressed spectral changes compared to the experiment with BAX in solution, suggesting a similar kind of interaction in the two cases. This provides further indication that interaction of BNIP3 with mitochondria takes place viaan interaction with mitochondrial BAX. In an attempt to substantiate this hypothesis, we used mitochondria lacking BAX, isolated from *Bax^−/−^* mouse hearts, for the EPR experiments (Figure 4E, 4th panel). Surprisingly, with *Bax^−/−^* mitochondria, the EPR spectrum changes even more considerably than with wt mitochondria, with more expressed mobile (m) and immobile (i) spectral components (see Figure 4E). This suggests that interaction of BNIP3 with mitochondria not only takes place via dimerization with BAX in the MOM, but also through another mechanism—either by direct interaction/insertion into the outer membrane or by interaction with another protein in the MOM. This notion is supported by a recent study using co-immunoprecipitation and mass spectrometry to show that BNIP3 interacts with VDAC in the MOM in SH-SY5Y human neuroblastoma cells [14]. Interestingly, for mitochondria lacking BNIP3, isolated from *Bnip3^−/−^* mouse hearts, EPR spectral changes are also observed (Figure 4E, bottom panel). In this case, the ratio between mobile and immobile components is shifted more toward the mobile fraction.

**Figure 5:**
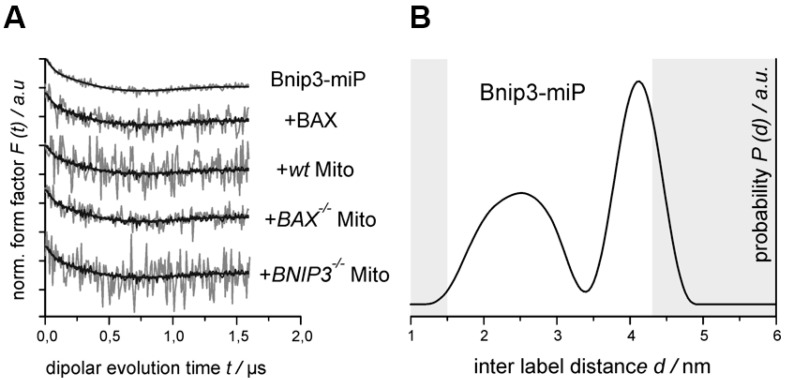
Double-electron-electron resonance (DEER) inter spin distance measurements. (**A**) Background corrected DEER dipolar evolution traces for (from top to bottom) rBNIP3-miP, rBNIP3-miP + recombinant BAX (rBAX), rBNIP3-miP + wt mitochondria, and with *Bax^−/−^* and *Bnip3^−/−^* mitochondria, with fits (thick lines) obtained by Tikhonov regularization (*n* = 2); (**B**) Inter spin distance distribution for rBNIP3-miP. The grey shaded areas indicate parts of the distance distribution that are not reliable due to experimental limitations (*n* = 2).

Single-site labeling of rBNIP3 results in the introduction of two labels in a BNIP3 homodimer; these are most likely symmetry-related. Thus, dipolar spectroscopy, namely (four pulse) double-electron-electron resonance (DEER, *aka* PELDOR) [23,24], can be applied to measure distances between these labels. Figure 5A (top) shows the background corrected dipolar evolution trace (form factor *F*(*t*)) together with the fit obtained by Tikhonov regularization (see Materials and Methods), and Figure 5B shows the corresponding distance distribution (*P*(*t*), right panel) for rBNIP3-miP in solution. As expected, a dipolar modulation is observed (Figure 5, left panel) that can be translated into a distance distribution (right panel), corroborating the presence of BNIP3 homodimers in solution. The observed distance distribution is broad, indicating conformational heterogeneity of rBNIP3 homodimers. This result appears to be well in line with the notion that most BH3-only proteins are unstructured in the absence of binding partners [25,26] and, thus, belong to the class of intrinsically disordered proteins (IDPs) [27,28]. IDPs do not have a well-defined tertiary structure and usually adopt a unique structure only upon, for example, binding to their cognate partners [29]. In case of the BNIP3 homodimer, its structural plasticity might be necessary to permit the interaction with its binding partner (BAX, VDAC, BCL-2) that appears—at least in the case of BAX—to replace the second BNIP3 protomer to form a heterodimer.

In the presence of a 25% molar excess of BAX, the obtained form factor (Figure 5A, 2nd row, gray) is identical to that for BNIP3 alone within the given experimental uncertainties. Thus, the interaction with BAX only takes place by “replacing” the second BNIP3 molecule with either one or two BAX molecules. In this case, BNIP3 molecules in both dimers and trimers with BAX are “invisible” for the DEER experiment (no second label in the oligomer) and the resulting signal arises solely from the remaining BNIP3dimer. Or, the trimers do comprise two BNIP3 moieties, but binding to BAX induces no conformational changes that would be reflected in a significant distance change between the labels. Based on the observed weak but reproducible changes in the cw EPR spectrum compared to that of BNIP3 alone (cf. Figure 4E, 2nd row) and our co-immunoprecipitation data (cf. Figure 3B,C) we favor the first explanation, as we would expect the cw spectral changes—that indicate an altered spin label mobility—to be also reflected in an altered inter label distance distribution.

To achieve first insights into the physiological BNIP3/BAX interaction in mitochondria, we also performed the DEER experiment with rBNIP3-miP in the presence of mitochondria isolated from wt mouse heart cells (Figure 5A, 3rd row). Also in this case, the DEER trace does not show differences to that for BNIP3 alone within the experimental error, and can be fitted satisfactorily assuming the same distance distribution (Figure 5B). Performing the analogous experiments with mitochondria isolated from *Bnip3^−/−^* and *Bax^−/−^* mice (Figure 5A, last and second-last row, respectively) yields the same results. These findings strongly suggest, that the BNIP3 interaction with mitochondria (and BAX) takes place with “monomeric” BNIP3, i.e., no BNIP3 dimers form complexes with BAX or other components of the MOM. Nevertheless, based on our current data, we cannot fully exclude that such interactions (e.g., BNIP3_2_/BAX) take place without affecting the distance distribution between the two spin labels. Experiments using the method of site-directed spin labeling to label selected positions in BNIP3 (and BAX), including introduction of two labels in each protomer to measure *intra* molecular distances, as well as more sophisticated experimental protocols to obtain the “pure” oligomeric species, will help to clarify this issue.

## 3. Materials and Methods

All chemicals were purchased from Sigma-Aldrich (Munich, Germany) unless otherwise noted.

### 3.1. Animals

C57BL/6 wild-type (wt) mice and B6.129X1-*Bax^tm1Sjk^*/J(*Bax^−/−^*) mice (12 ± 3 weeks) were obtained from Janvier (Saint Berthevin, France) and the Jackson Laboratory (Bar Harbor, ME, USA) and kept for one week in the local animal house for acclimatization. C57BL/6J-TgH (*Bnip3^−/−^*) mice (12 ± 3 weeks) were bred at the animal facility of the University Hospital Essen. The mice were killed by cervical dislocation and the hearts were directly excised for mitochondria preparation. All animal experiments were performed after obtaining relevant permission (23 February 2015) according to the “European Convention for the Protection of Vertebrate Animals used for Experimental and other Scientific Purposes” (EU Directive 2010/63/EU).

### 3.2. Induction of In Vivo Ischemia

The induction of ischemia was performed as described before [30,31,32]. Briefly, wt mice were anesthetized by intraperitoneally injection of ketamine (100 mg/kg) and xylazine (10 mg/kg) and intubated. Mechanical ventilation parameters were set to a tidal volume of 2.1 to 2.5 mL and a respiratory rate of 140 breaths per min using a mouse mini-ventilator. Deep anesthesia was maintained by adding 2 vol % isoflurane to the ventilation gas. The chest was opened through a lateral thoracotomy (1 cm left lateral incision between the 3rd and 4th rib). A 6-0 prolene suture was placed around the left coronary artery (LCA) and a piece of soft silicon tubing was placed over the artery. Coronary occlusion was achieved by tightening and tying the suture. After 30 min occlusion and flushing out the blood with a sodium chloride solution, hearts were excised.

### 3.3. Recombinant BNIP3 and BAX

Murine recombinant BNIP3 (rBNIP3) was a generous gift from Raphael Stoll (Ruhr-University Bochum, Germany). Murine recombinant BAX (rBAX) was obtained from MyBioSource (MBS555502).

### 3.4. Preparation of Mouse Heart Mitochondria

Extracted hearts from wt, *Bnip3^−/−^* and *Bax^−/−^* mice were chopped manually in small pieces and washed three times blood-free in an ice-cold homogenization buffer containing 250 mM sucrose, 10 mM 2-(4-(2-Hydroxyethyl)-1-piperazinyl)-ethansulfonsäure (HEPES), 1 mM ethylene glycol-bis(β-aminoethyl ether)-*N*,*N*,*N*′,*N*′-tetraacetic acid (EGTA) (pH 7.4). The tissue was homogenized in 4 mL homogenization buffer containing 0.5% bovine serum albumin using an Ultra-Turrax (IKA-Werke, Staufen im Breisgau, Germany), centrifuged for 10 min at 700× *g*, 4 °C, to remove unbroken tissue and nuclei. Supernatant was centrifuged for 10 min at 15,000× *g*, 4 °C to pellet mitochondria. The mitochondria pellet was washed twice with homogenization buffer. To obtain cytosol, the supernatant was centrifuged for 30 min at 105,000× *g*, 4 °C.

### 3.5. Incubation of rBNIP3 with Mitochondria

Isolated mouse heart mitochondria (250 µg) were incubated with 10 µM rBNIP3 for 10 min, room temperature (RT), in homogenization buffer. Mitochondria were centrifuged for 5 min at 12,700× *g*, 4 °C and lysed for immunoblot analyses to measure BNIP3 protein concentration.

### 3.6. Alkali Extraction

To analyze the intensity of the BNIP3 and mitochondria interaction, we performed alkali extraction. Isolated mouse heart mitochondria were resuspended in ice cold 0.1 M Na_2_CO_3_ (pH 11.5) and incubated for 20 min on ice. Membranes were recovered by centrifugation for 45 min at 20,000× *g*, 4 °C and lysed for immunoblot analyses (integral membrane proteins). Proteins in supernatant were concentrated using Centrifugal Filter Units (Millipore, Darmstadt, Germany) (soluble membrane proteins). Both fractions were analyzed by immunoblotting.

### 3.7. Flow Cytometry Analysis

Isolated mitochondria were resuspended in FACS-buffer, containing 220 mM sucrose, 68 mM mannitol, 10 mM KCl, 5 mM KH_2_PO_4_, 2 mM MgCl_2_, 500 µM EGTA, 5 mM succinate, 10 mM HEPES pH 7.2, 0.1% bovine serum albumin, and protease- and phosphatase inhibitors, and incubated with a primary antibody against BNIP3 (Abcam, Cambridge, UK) for 30 min, 4 °C. After washing twice with FACS-buffer, mitochondria were incubated with a secondary antibody labeled with AF488 for 30 min, 4 °C. After washing with FACS-buffer, the organelles were incubated with 1 µM MitoTrackerOrange (ThermoFisher Scientific, Schwerte, Germany), whose accumulation in mitochondria is dependent upon membrane potential, for 30 min, 4 °C. Mitochondria were then analyzed by flow cytometry using FACS Verse (Becton Dickinson, Heidelberg, Germany) [33,34]. PE Texas Red signal detector with excitation wavelength of 496 nm and emission of 615 nm were used to detect MitoTracker. To detect BNIP3, we used the FITC detector with excitation wavelength of 495 nm and emission wavelength of 519 nm.

### 3.8. Immunoblotting Analyses

Hearts were lysed in a buffer containing 50 mM Tris-HCl, 150 mM NaCl, 0.5 mM EDTA, 1% NP-40, and protease- and phosphatase inhibitor (pH 7.4). Isolated mitochondria were lysed in a buffer containing 200 mM sucrose, 10 mM HEPES, 1 mM EGTA, 1% Triton-X100 and protease- and phosphatase inhibitor (pH 7.4). The lysates were cleared by centrifugation (15,000× *g*, 15 min, 4 °C). Protein concentration in supernatant was measured using DC Protein Assay (Bio-Rad, Munich, Germany). Equivalent amounts of protein were separated by SDS/PAGE using 4%–12% Bis-Tris gels (ThermoFisher Scientific), transferred to nitrocellulose, and immunoblotted with primary antibodies against ANT1, BAX, BNIP3, and Tubulin (Abcam). The secondary antibodies used were horseradish peroxidase conjugated goat anti-mouse or anti-rabbit IgG (ThermoFisher Scientific). Immunoblotting was detected by ECL (ThermoFisher Scientific) and imaged on an Imager 600 (GE Healthcare, Freiburg, Germany) [35].

### 3.9. Co-Immunoprecipitation

Immunoprecipitation were performed using protein G-coupled dynabeads (ThermoFisher Scientific) [36]. Lysed proteins (500 µg) were incubated with 2 µg BNIP3/BAX antibody over night at 4 °C with shaking in a PBS buffer containing 1 mM DTT, 0.005% Brij35 and protease-phosphatase inhibitors. After 24 h, 20 µL dynabeads were added and the solution was incubated again for 1 h. The precipitated immune complex was washed twice and then resuspended in an elution buffer containing lithium dodecyl sulfate (LDS)-Sample Buffer (1:4) and Reducing Agent (1:10) (ThermoFisher Scientific) in PBS and heated for 5 min at 95 °C. After removal of the dynabeads, the eluate was analyzed via immunoblotting.

### 3.10. Transmission Electron Microscopy

Isolated mitochondria were fixed by adding double concentrated fixative (2% glutaraldehyde, 8% formaldehyde mixture in 0.1 M phosphate buffer, pH 7.4) at a 1:1 ratio to the mitochondrial suspension. After 10 min, organelles were spun into a pellet and fixation was continued with fresh fixative (1% glutaraldehyde, 4% formaldehyde in 0.1 M phosphate buffer, pH 7.4) for 1 h. After fixation, organelles were centrifuged to a pellet, washed with PBS (3 × 10 min) and H_2_O (2 × 10 min), and embedded in 2.5% agarose in distilled water. Agarose pellets containing mitochondria were postfixed in 1% osmiumtetroxide, dehydrated in acetone, and embedded in Epon LX 112 (Ladd Research Industries, Williston, VT, USA). Thin sections were cut with Leica Ultracut ultramicrotome, stained in uranyl acetate and lead citrate, and examined in a Tecnai G2 Spirit transmission electron microscope (FEI Europe, Eindhoven, The Netherlands). Images were captured using a Quemesa charge-coppled device (CCD) camera (Olympus Soft Imaging Solutions GmbH, Munster, Germany).

### 3.11. Heart Viability Assay

Mice were killed by cervical dislocation, and extracted mouse hearts were washed blood-free via aortic perfusion with an ice cold NaCl solution. The tissue was wrapped in a clear food wrap and stored for one hour in a −20 °C freezer. The heart was then serially sectioned perpendicularly to the long axis in 1-mm slices. The sections were incubated in 1% TTC for 5 min at 37 °C for demarcation of the viable (red) and non-viable (white) myocardium.

### 3.12. Spin Labeling of rBNIP3 with miPROXYL

The spin label 3-Maleimido-2,2,5,5-tetramethyl-1-pyrrolidinyloxy (3-Maleimido-PROXYL, miPROXYL) was covalently attached to the conserved cysteine residue of rBNIP3. Then, the protein in buffer (PBS, pH 7.4, with 0.005% Brij35) was incubated with 10 mM DTT for 2 h. DTT was removed by repeated buffer exchanges using a 50 mM PBS buffer, pH 7.4. Subsequently, the protein was incubated for 40 h at 4 °C with 2 mM miPROXYL. Excess label was also removed by repeated washing steps using the same buffer. The spin-labeling efficiency was 70% and has been determined by double integration of room temperature cw spectra and comparison with a reference sample of known spin concentration. For the DEER (double electron-electron resonance) measurements at low temperature (50 K), the buffer was supplemented with 25% glycerol (*v*/*v*).

### 3.13. EPR Spectroscopy

Cw EPR spectra at room temperature (298 K) were recorded with a home-made EPR spectrometer equipped with a Bruker dielectric resonator (MD5), with the microwave power set to ~0.5 mW and a B-field modulation amplitude of 1.5 G. Samples (20 µL) were loaded into EPR glass capillaries with a 0.9 mm inner diameter.

DEER distance measurements were performed at Q-band frequencies (~34 GHz) with a BrukerElexsys 580 spectrometer equipped with a Q-band bridge and a 150 W Q-band travelling wave tube (TWT) amplifier using a BrukerFlexline resonator ER 5106QT-2. The temperature was stabilized at 50 K using a continuous flow helium cryostat ESR900 (Oxford Instruments, Abingdon, Oxfordshire, UK) controlled by an Oxford Intelligent temperature controller ITC 503S. Measurements were performed using the four-pulse DEER sequence [23,24]:

π/2 (*ν*_obs_) − τ_1_ − π (*ν*_obs_) − *t’* − π (*ν*_pump_) − (τ_1_ + τ_2_ − *t’*) − π (*ν*_obs_) − τ_2_ − echo

A two-step phase cycling (+〈x〉, −〈x〉) was performed on π/2 (ν_obs_). Time *t’* is varied, whereas τ_1_ and τ_2_ are kept constant. The dipolar evolution time is given by *t* = *t’* − τ_1_. Data were analyzed only for *t* > 0. The resonator was overcoupled to Q ≈ 100; the pump frequency *ν*_pump_ was set to the center of the resonator dip, coinciding with the maximum of the nitroxide EPR spectrum, whereas the observer frequency *ν*_obs_ was 50 MHz higher. All measurements were performed with observer pulse lengths of 16 ns for π/2 and 32 ns for π pulses and a pump pulse length of 32 ns. Proton modulation was averaged by adding traces at eight different τ_1_ values, starting at *τ*_1,0_ = 320 ns and incrementing by ∆*τ*_1_ = 16 ns. Data points were collected in 8-ns time steps. The total measurement time for each sample was 24–48 h. Data analysis was performed with the software package DeerAnalysis [37].

## 4. Conclusions

BNIP3 is a regulator of biological processes in pathology [9], and it has been shown to interact with numerous mitochondrial and non-mitochondrial proteins like BCL-2, VDAC, BCL-X_L_, and OPA1 in different cells. Furthermore, recent studies provided hints that BAX is a downstream effector of BNIP3-mediated cell death [1,38]. Unexpectedly, our results reveal that under physiological conditions BNIP3 forms reduction-resistant heterodimers with BAX in the MOM. This proves a direct interaction of the two proteins in vivo in absence of pathophysiological stimuli (e.g., hypoxia, acidosis). However, we found that cardiac BNIP3 is not exclusively associated with mitochondria under basal conditions. We further identified that BNIP3 is also located within the cytosol of adult mouse heart cells, where it appears as dimers. Contrary to the MOM, no heterodimer with BAX is formed in the cytosol. Our findings further reveal that a fraction of BNIP3 dimers in the cytosol is held together by non-covalent bonds, and the BNIP3 interaction with mitochondria takes place between “monomeric” BNIP3 and mitochondrial BAX. This suggests that a replacement of one BNIP3 moiety of the cytosolic homo/heterodimer by mitochondrial BAX forms the heterodimer in the MOM. Our results show that cytosolic homo/heterodimerization of BNIP3 and interaction with mitochondria via mitochondrial BAX does not affect MIM potential and cell viability, unlike what was assumed to occur in cardiac disease. It is tempting to speculate that the formation of these dimers accounts for a regulatory control of cell death, blunting the ability to trigger mitochondrial-induced cell death that each molecule produces [22,39,40]. Strinkingly, BAX interactions with different anti-apoptotic BCL-2 proteins seem to be weakly. BNIP3/BAX dimerization might affect the activity of these proteins, including BCL-2 [41,42].
